# Therapeutic potential of the Nrf2 activator omaveloxolone in spinocerebellar ataxia type 3 in cellular and *Drosophila* models

**DOI:** 10.3389/fphar.2026.1872142

**Published:** 2026-08-05

**Authors:** Shin-Hung Pan, Jui-Chih Chang, Wan-Hsuan Lin, Fang-Hsin Leng, Wen-Ling Cheng, Wei-Yong Lin, Chin-San Liu

**Affiliations:** 1 Department of Vascular and Genomic Center, Institute of ATP, Changhua Christian Hospital, Changhua, Taiwan; 2 Center of Regenerative Medicine and Tissue Repair, Institute of ATP, Changhua Christian Hospital, Changhua, Taiwan; 3 General Research Laboratory of Research Department, Changhua Christian Hospital, Changhua, Taiwan; 4 College of Chinese Medicine, Graduate Institute of Integrated Medicine, China Medical University, Taichung, Taiwan; 5 Department of Neurology, Changhua Christian Hospital, Changhua, Taiwan

**Keywords:** autophagy, *Drosophila*, Nrf2, omaveloxolone, oxidative stress, spinocerebellar ataxia type 3

## Abstract

**Introduction:**

Spinocerebellar ataxia type 3 (SCA3), also known as Machado–Joseph disease, is an autosomal dominant polyglutamine neurodegenerative disorder caused by a CAG repeat expansion in the ataxin-3 gene (ATXN3). Mutant ataxin-3 accumulation, oxidative stress, mitochondrial dysfunction, and impaired protein quality control contribute to its pathogenesis; however, no disease-modifying therapy is currently available. Omaveloxolone (RTA-408), an activator of nuclear factor erythroid 2–related factor 2 (Nrf2), is approved for Friedreich’s ataxia, but its therapeutic potential in SCA3 remains unclear.

**Methods:**

We evaluated the effects of RTA-408 in MJD78 cells and Drosophila SCA3 models. Cell viability, apoptosis, mutant ataxin-3 levels, Nrf2-associated antioxidant proteins, p62/autophagy-related markers, and mitochondrial phenotypes were assessed in MJD78 cells. Survival, climbing ability, and external eye degeneration were evaluated in SCA3tr-Q78 flies.

**Results:**

In MJD78 cells, 0.3 and 0.5 μM RTA-408 more consistently improved cell viability, reduced apoptosis, and decreased detectable mutant ataxin-3 accumulation than 0.1 μM RTA-408. RTA-408 increased the nuclear-to-cytoplasmic Nrf2 ratio and upregulated NQO1, HO-1, and SOD2 without significantly altering intracellular or mitochondrial reactive oxygen species levels. RTA-408 also increased p62 expression, whereas ATG7, Beclin 1, LAMP2, and the LC3-II/LC3-I ratio showed limited or nonsignificant changes, indicating p62 upregulation without definitive evidence of canonical autophagic flux activation. In addition, 0.3 μM RTA-408 partially shifted mitochondrial morphology from globe-like to elongated forms and increased mitochondrial DNA copy number. In SCA3tr-Q78 flies, RTA-408 partially improved survival, locomotor performance, eye size, and pigmentation, with more evident benefits at earlier stages.

**Conclusion:**

These findings support the protective potential of RTA-408 in SCA3-related models. Its effects were associated with activation of Nrf2-related antioxidant responses, p62 upregulation, and selected improvements in mitochondrial phenotypes. Further studies are needed to clarify the underlying mechanisms and determine the translational potential of RTA-408 for SCA3.

## 1 Introduction

Spinocerebellar ataxias (SCAs) comprise a heterogeneous group of inherited neurodegenerative disorders characterized by progressive cerebellar ataxia, most commonly with autosomal dominant inheritance. Their global prevalence has been estimated to range from approximately 1–5 cases per 100,000 individuals, although prevalence varies by population and genetic subtype (Sullivan et al., 2019). Among polyglutamine (polyQ) disorders, type 3 SCA (SCA3), also known as Machado–Joseph disease (MJD), is one of the most prevalent and is considered second only to Huntington’s disease (McLoughlin et al., 2020). In a Taiwanese clinical cohort, SCA3 accounted for approximately 54.5% of genetically diagnosed SCA cases (Chen et al., 2019). SCA3 is caused by an expanded CAG repeat in exon 10 of ATXN3, resulting in an ataxin-3 protein with an elongated polyQ tract that undergoes misfolding and aggregation, thereby disrupting proteostasis networks in which ataxin-3 normally functions as a deubiquitination-related protein (Haacke et al., 2006; Burnett et al., 2003; Winborn et al., 2008). Normal alleles typically contain 12–44 CAG repeats, whereas pathogenic alleles are often in the ∼60–97 range and inversely correlate with age at onset (McLoughlin et al., 2020). Despite extensive efforts, disease-modifying therapies for SCA3 remain unavailable (Sullivan et al., 2019).

Emerging evidence indicates that SCA3 pathogenesis extends beyond protein aggregation to involve convergent cellular stress pathways, including oxidative stress, mitochondrial dysfunction, and impaired protein quality-control systems (McLoughlin et al., 2020; Harmuth et al., 2022; Paulino and Nobrega, 2023; Watchon et al., 2023). These processes are highly interconnected: mutant ataxin-3 accumulation perturbs redox homeostasis and mitochondrial integrity, while defective proteostasis amplifies cellular vulnerability and neurodegenerative cascades.

Nuclear factor erythroid 2–related factor 2 (Nrf2; encoded by NFE2L2) is a master regulator of cytoprotective stress responses that controls antioxidant gene expression and interacts with proteostasis- and mitochondrial stress-related networks (Wu et al., 2022; Wu et al., 2018; Chang and Chen, 2024). Omaveloxolone (RTA-408), a second-generation oleanane triterpenoid Nrf2 activator, has demonstrated clinical efficacy in Friedreich’s ataxia, improving mitochondrial and redox phenotypes and conferring neurological benefit (Lynch et al., 2021; Food U and Administration D, 2023), supporting its therapeutic potential in related neurodegenerative conditions. Beyond Friedreich’s ataxia, RTA-408 and related Nrf2-activating triterpenoids have been investigated in preclinical disease models involving oxidative stress, inflammation, and mitochondrial stress, providing a broader pharmacological rationale for testing this compound in neurodegenerative disease contexts (Wu et al., 2022; Wu et al., 2018; Lynch et al., 2021; Wu et al., 2024; Ulasov et al., 2022).

Our previous work showed that the Nrf2 activator curcumin reduces mutant ataxin-3 accumulation in MJD78 cells through enhancement of protein clearance-related mechanisms (Wu et al., 2022). However, whether similar effects are involved in RTA-408 regulation remains unclear. Thus, we evaluated RTA-408 in cellular and *Drosophila* models of SCA3, focusing on Nrf2-associated antioxidant responses, p62/autophagy-related protein readouts, selected mitochondrial phenotypes, and disease-relevant functional outcomes.

## 2 Materials and methods

### 2.1 Cell culture and treatment

The human neuroblastoma cell line (SK-N-SH) stably expressing the full-length human ATXN3 gene containing 26 (MJD26) or 78 CAG (MJD78) repeats was provided by Dr. Mingli Hsieh (Department of Life Sciences, Tunghai University, Taiwan) and maintained as previously described in our previous SCA3 cellular studies (Wu et al., 2022; Wu et al., 2018). Cells were cultured in Dulbecco’s modified Eagle’s medium (DMEM; GIBCO, Grand Island, NY, USA) supplemented with 1% penicillin/streptomycin (GIBCO), 1% L-glutamine (GIBCO), 1% non-essential amino acid (NEAA; GIBCO) and 10% heat-inactivated fetal bovine serum (FBS; GIBCO) at 37 °C in a humidified incubator containing 5% CO2. The culture medium was replaced every 2 days and cells were passaged once weekly. For drug treatment, cells were exposed to omaveloxolone (RTA-408; HY-12212,10mg, MedChemExpress) at final concentration of 0, 0.1, 0.3, or 0.5 μM for 24 h. The concentrations of 0.1, 0.3, and 0.5 μM were selected to examine a low-submicromolar range based on prior experience with Nrf2 activator treatment in this SCA3 cellular model and previously published RTA-408 treatment conditions in MJD78 cells (Wu et al., 2022).

### 2.2 WST-1 assay for cell viability

Cell viability was assessed using the Cell Proliferation Reagent Water Soluble Tetrazolium Salt-1 (WST-1; 11644807001, Roche, Mannheim, Germany) according to the manufacturer’s instructions. MJD26 and MJD78 cells were seeded in 96-well plates at a density of 2 × 104 cells/well. After 24 h of attachment, the culture medium was replaced and the cells were treated with or without RTA-408 for 24 h. WST-1 reagent was then added at 10 μL per 100 μL culture medium, and the cells were incubated at 37 °C for 3h. Absorbance was measured at 450 and 600 nm using a CLARIOstar® high-performance monochromator multimode microplate reader (BMG labtech, Ortenberg, Germany). Cell viability analysis was performed in five independent experiments.

### 2.3 Annexin V/7-AAD assay for apoptosis

Apoptosis was evaluated by Annexin V and 7-aminoactinomycin D (7-AAD) staining using the FlowCellect® MitoDamage Kit (FCCH100106, Millipore, Billerica, MA, USA), followed by analysis on a NucleoCounter® NC-3000 fluorescence advanced image cytometer (ChemoMetec, Allerød, Denmark). MJD26 and MJD78 cells were seeded in 12-well plates at a density of 4 × 105 cells/well. After 24 h of treatment, cells were harvested and resuspended in 1× assay buffer HSC containing Annexin V and 7-AAD to identify apoptotic and dead-cell populations, respectively. The samples were loaded onto NC-slide A2 (ChemoMetec) and immediately analysed. Apoptosis analysis was performed in seven independent experiments.

### 2.4 Measurement of intracellular ROS, mitochondrial ROS

Intracellular reactive oxygen species (ROS) and mitochondrial ROS were measured using CM-H2DCFDA and MitoSOX Red (M36008, Thermo Fisher Scientific, Waltham, MA, USA), respectively, and analyzed using the NucleoCounter® NC-3000 (ChemoMetec). Intracellular ROS and mitochondrial ROS analysis was performed in four independent experiments.

### 2.5 Analysis of mitochondrial morphology

For mitochondrial visualisation, cells were seeded in μ-Slide 8 well chamber slides (Ibidi GmbH, Munich, Germany) and treated with RTA-408 for 24 h. Cells were then stained with MitoTracker Green (250 nM; Invitrogen, Carlsbad, CA, USA) at 37 °C in 5% CO2 for 30 min, followed by washing with culture medium. Subsequently, cells were incubated with Hoechst 33342 (1:2000 dilution in culture medium) at 37 °C in 5% CO2 for 5 min and washed again with medium. Fluorescence images were acquired using FLUOVIEW FV 3000 Confocal Laser Scanning Microscope (Olympus, Tokyo, Japan). Mitochondrial morphology was classified using the automated morphological subtyping system, MicroP, described by Peng et al. and classified into globe forms, including swollen globes (yellow) and small globes (blue); tube forms, including straight tubules (green); and branching tube forms, including branched tubules (purple), twisting tubules (orange), and loops (red) (Peng et al., 2011). Three independent experiments were performed; in each group, three independent microscopic fields were analyzed, and approximately 250–350 mitochondrial objects derived from about 4–6 cells per image were quantified.

### 2.6 Mitochondrial DNA copy number analysis

Total cellular DNA was extracted from MJD26 and MJD78 cells using the Gentra Puregene DNA kit (Qiagen, Hilden, Germany). A total of 40 ng of DNA was subjected to fluorescence-based quantitative polymerase chain reaction (qPCR) to amplify the mitochondrial gene (MT-ND1) and the nuclear reference gene (β-globin). The primer sequences for MT-ND1 were 5′-AAC​ATA​CCC​ATG​GCC​AAC​CT-3’ (forward) and 5′-AGC​GAA​GGG​TTG​TAG​TAG​CCC-3’ (reverse). The primer sequences for β-globin were 5′-GAA​GAG​CCA​AGG​ACA​GGT​AC-3’ (forward) and 5′-CAA​CTT​CAT​CCA​CGT​TCA​CC-3’ (reverse). The relative copy number of mitochondrial DNA (mtDNA) was estimated from the threshold cycle (Ct) values of these two genes. The intra-assay coefficient of variation was 10.29%. mtDNA copy number analysis was performed in three independent biological experiments, and qPCR reactions were run in technical duplicates.

### 2.7 Western blotting

For whole-cell lysates, cells were lysed in RIPA buffer (50 mM Tris pH7.5, 150 mM NaCl, 10 mM EDTA, 1% NP-40, 0.1% SDS) supplemented with proteinase inhibitor cocktail (Sigma-Aldrich, St. Louis, MO, USA) and phosphatase inhibitor Cocktail Set V (Millipore). Protein concentrations were determined using the PierceTM BCA Protein Assay Kit (Thermo Fisher Scientific). For Nrf2 fractionation analysis, nuclear and cytoplasmic proteins were extracted using a commercial cytoplasmic and nuclear protein extraction kit according to the manufacturer’s instructions. Whole-cell lysates and nuclear fractions were quantified by BCA assay (Pierce, Rockford, IL, USA). Equal amounts of denatured proteins (20 μg) were separated by SDS-PAGE and transferred onto PVDF membranes (Millipore). Membranes were blocked with EveryBlot Blocking Buffer (BIO-RAD, Hercules, CA, USA) for 1h and incubated with primary antibodies against ataxin-3, ATG7, p62, LAMP2, Beclin 1, Nrf2, NQO1, LC3, HO-1, SOD1, SOD2, catalase. Immunoreactive bands were detected using horseradish peroxidase-conjugated secondary antibodies (Jackson ImmunoResearch, West Grove, PA, USA) and enhanced chemiluminescence (ECL) reagents (WBKLS0500, Millipore). Band intensities were quantified using ImageJ. For whole-cell Western blot analyses, target protein signals were normalized to the total protein signal of each lane. Whole-lane total protein normalization was used for whole-cell Western blotting to reduce loading bias across lanes, and uncropped raw blot images with total protein signals are provided in Supplementary Figure S1. Densitometric quantification was performed using the original image files before figure assembly, and the representative blots shown in the figures were not used as the sole basis for quantification. For nuclear/cytoplasmic Nrf2 analysis, glyceraldehyde 3-phosphate dehydrogenase (GAPDH; Millipore), Lamin B and Histone H3 (Abcam) were used as the cytoplasmic and nuclear reference markers, respectively. Nuclear/cytoplasmic Nrf2 analysis was performed in three independent experiments, and all other Western blot analyses were performed in four independent experiments.

All primary antibodies were obtained from the indicated commercial sources and detailed antibody information including host species, catalogue number, dilution, and RRID is provided in Supplementary Table S1.

### 2.8 *Drosophila* stocks and crosses

The UAS-SCA3tr-Q27, UAS-SCA3tr-Q78 (w), elav-Gal4 and gmr-Gal4 fly strains were obtained from the Bloomington *Drosophila* Stock Centre (Indiana University, Bloomington, IN, USA). Flies were maintained on standard cornmeal medium at 25 °C and 60%–70% relative humidity under a 12h light/12h dark cycle. F1 progeny expressing ataxin-3tr-Q27 or ataxin-3tr-Q78 in the nervous system were generated by crossing virgin females carrying elav-Gal4 or gmr-Gal4 on the X chromosome with males carrying UAS-Q27 or UAS-Q78. After eclosion, elav-SCA3tr-Q27 or gmr-SCA3tr-Q27 flies reared on DMSO-containing medium served as controls. elav-SCA3tr-Q78 or gmr-SCA3tr-Q78 flies were randomly assigned to treatment groups receiving DMSO alone or RTA-408 at 1, 2 or 4 μM. In the elav-driven model, mutant ataxin-3 was expressed in the nervous system to assess adult survival and locomotor decline. In the gmr-driven model, mutant ataxin-3 was expressed in developing eye tissues to assess external eye degeneration, eye size, pigmentation, and ommatidial morphology. Consistent with our previous SCA3 *Drosophila* studies, female flies were used in the main experiments (Wu et al., 2024). In preliminary experiments of the present study, male and female flies were assessed separately, and males showed a markedly shorter post-eclosion lifespan and less stable phenotypic responses; therefore, female flies were used in all subsequent experiments.

### 2.9 Standard fly food preparation

Flies were maintained on standard cornmeal medium. For drug treatment, RTA-408 was dissolved in DMSO and added to cooled fly food to final concentrations of 1, 2, or 4 μM. The same final concentration of DMSO was used in control and treatment food. Drug-containing food was dispensed into vials, stored at 4 °C, and used within 1 month.

### 2.10 Survival assay

After eclosion, 3-day-old elav-SCA3tr-Q27/78 flies were transferred to vials containing standard food supplemented with DMSO or RTA-408 (1, 2 and 4 μM). At the beginning of the experiment, each group contained 350 flies distributed in 10 vials, with 35 flies per vial. No formal *a priori* power analysis was performed. The sample size of 35 flies per vial and 10 vials per group was selected based on our previous SCA3 *Drosophila* studies and commonly used survival assay designs, while maintaining manageable vial density and reducing overcrowding-related stress. Dead flies were counted and the surviving flies were transferred to fresh food every 3 days. Survival curves were analysed using the Kaplan-Meier method and compared among groups using the log-rank test in SigmaStat version 3.5 (Systat Software, San Jose, CA, USA). The assay design was adapted from previously described *Drosophila* survival and behavioural paradigms with modifications for the present SCA3 model (Wu et al., 2017).

### 2.11 Climbing assay

The climbing ability was assessed every 3 days using a negative geotaxis assay. In each batch, 35 flies per group were tested. After being gently tapped to the bottom of an empty vial, flies were allowed to climb for 18 s, and the number of flies that climbed above the 5 cm mark was recorded. Climbing activity (%) was calculated as the percentage of flies that climbed above the 5 cm line within the testing period. Each group was tested three times per session, and the highest value was used for analysis according to a predefined rule intended to reduce the influence of transient handling-related inactivity, delayed initiation of climbing, and random disturbance during repeated trials. A total of eight independent batches were performed (Wu et al., 2017; Sofola et al., 2010). Because the total number of surviving flies could vary among vials and across time points owing to mortality, proportional data were transformed using the arcsine square-root transformation before statistical analysis to reduce bias associated with unequal denominators. This assay format followed established *Drosophila* negative geotaxis paradigms, with minor modifications in climbing distance, time window, and replicate structure for the present study.

### 2.12 Scanning electron microscopy

For scanning electron microscopy (SEM), adult female fly heads from the selected experimental groups were briefly immersed in 2% PBST to reduce surface tension and remove surface debris, and were then transferred to 1.5-mL microcentrifuge tubes containing 4% paraformaldehyde for fixation overnight at 4 °C (Poole et al., 2008). On the following day, samples were dehydrated on ice through a graded ethanol series consisting of 10%, 30%, 50%, 70%, and 85% ethanol for 15 min each, followed by three changes of 95% ethanol for 30 min each and two changes of absolute ethanol for 30 min each. After dehydration, samples were placed in 6 cm dishes and air-dried overnight under laminar flow. Dried heads were then mounted on SEM stubs, sputter-coated with gold, and imaged using a HITACHI S-3400N scanning electron microscope. SEM imaging was performed by the Taxonomy and Ecology of Forest Plant Laboratory, Department of Forestry, National Chung Hsing University, Taichung, Taiwan. Three eyes were analyzed per group.

### 2.13 Eye morphology, eye size, and pigmentation analysis

Heads from 6- and 20-day-old female flies were mounted and imaged under an Olympus BX51 optical microscope (Tokyo, Japan) using the same magnification and imaging settings across groups. Eight eyes per group were analyzed. Eye boundaries were manually defined in ImageJ according to the visible compound eye margin, and the same workflow was applied to all images to maintain consistency of region-of-interest selection. To quantify eye degeneration in gmr-SCA3tr-Q27 and gmr-SCA3tr-Q78 flies, the area of remaining normal pigmentation was measured in ImageJ as follows: green-channel extraction to generate greyscale images; definition of the compound eye as the region of interest; Gaussian blur smoothing and binary thresholding using consistent criteria; and calculation of the integrated intensity of the remaining normal pigment within the region of interest (Saitoh et al., 2015).

### 2.14 Statistical analysis

Except for the flies survival assay, all data in the present study were analysed using one-way analysis of variance (ANOVA) followed by Tukey’s honestly significant difference (HSD) *post hoc* test in R version 4.5.0 (Team, 2025). For the climbing assay, the proportional data were transformed into square-roots of arcsine before statistical analysis. Survival curves were analysed using the Kaplan-Meier method and compared using the log-rank test. Data are presented as mean ± standard deviation (SD), and a two-sided P < 0.05 was considered statistically significant. Formal blinding was not applied to all experiments. However, instrument-based or predefined analytical workflows were used for quantitative analyses, including absorbance-based viability measurement, NC-3000 fluorescence assays, ImageJ densitometry, MicroP mitochondrial morphology classification, and ImageJ-based eye area and pigmentation quantification.

## 3 Results

### 3.1 RTA-408 improves cell viability, suppresses apoptosis, and reduces mutant ataxin-3 accumulation

**FIGURE 1:**
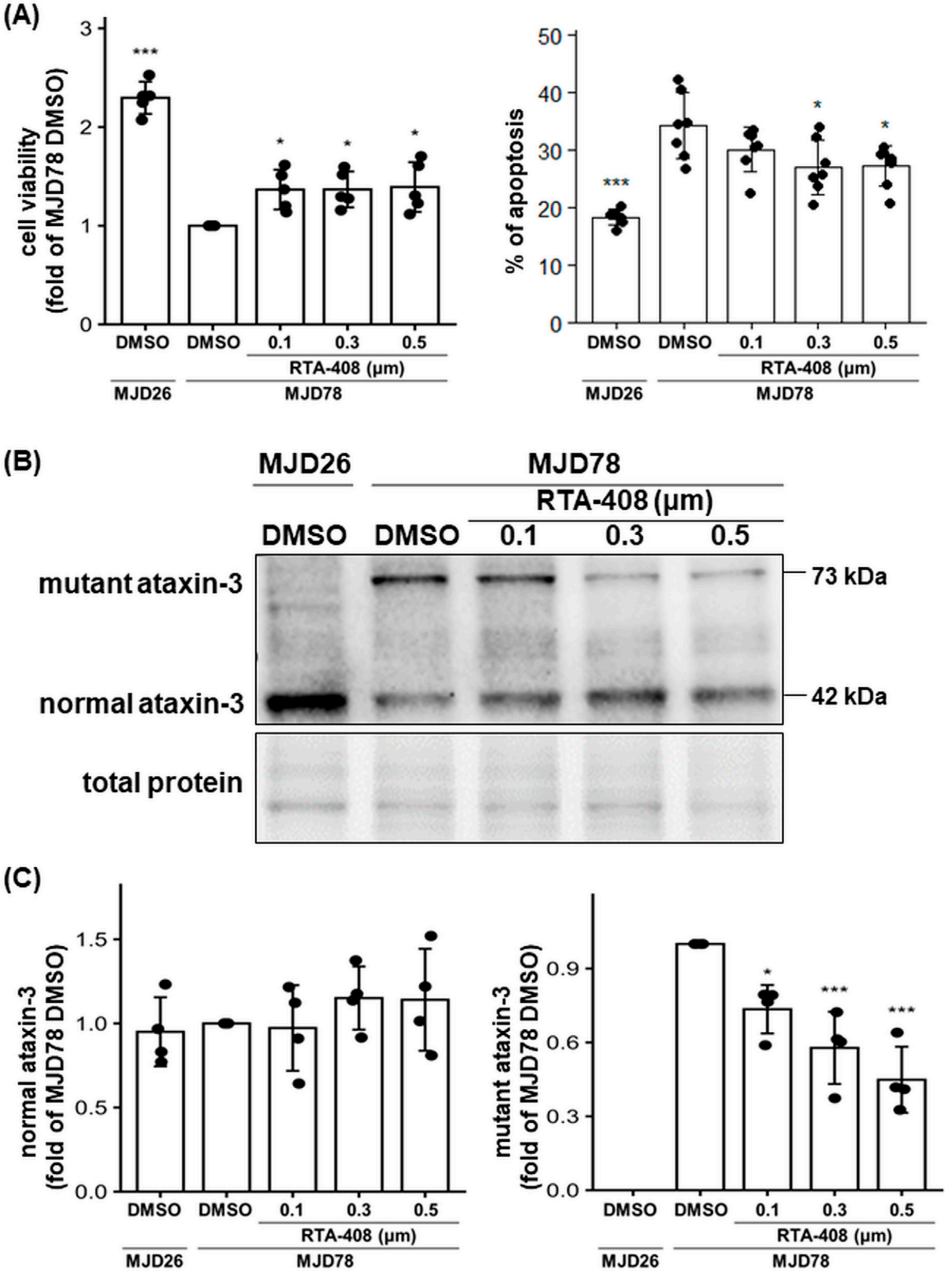
RTA-408 improves viability and reduces apoptosis while decreasing mutant ataxin-3 in MJD78 cells. **(A)** Cell viability measured by WST-1 assay and apoptosis measured by Annexin V/7-AAD staining in MJD26 and MJD78 cells treated with DMSO or RTA-408 (0.1, 0.3, or 0.5 μM) for 24 h. **(B)** Representative Western blot of normal and mutant ataxin-3. Total protein was used as the loading reference. The uncropped raw Western blot images from independent experiments are provided in Supplementary Figure S1. **(C)** Densitometric quantification of normal and mutant ataxin-3, normalized to total protein and performed using the original image files before figure assembly. Data are mean ± SD; WST-1, n = 5; apoptosis, n = 7; Western blot, n = 4 independent experiments. One-way ANOVA with Tukey’s HSD *post hoc* test. Asterisks indicate statistical significance vs. MJD78 + DMSO: ***: P < 0.001, **: P < 0.01, *:P < 0.05. RTA-408: omaveloxolone.

Compared with MJD26 control cells, MJD78 cells treated with DMSO exhibited reduced cell viability and an increased apoptotic fraction. Treatment with RTA-408 (0.1–0.5 μM) improved cell viability in a dose-dependent manner, with the most pronounced effect observed at 0.5 μM, and significantly reduced apoptosis at 0.3 and 0.5 μM (Figure 1A). At the protein level, mutant ataxin-3 was readily detected in MJD78 cells and was reduced following RTA-408 treatment in a dose-dependent manner, whereas normal ataxin-3 levels remained largely unchanged (Figures 1B,C). These results indicate that RTA-408 provides cellular protection in MJD78 cells and reduces detectable mutant ataxin-3 accumulation under the present experimental conditions.

### 3.2 RTA-408 increases p62 expression without clear activation of canonical autophagy markers

**FIGURE 2:**
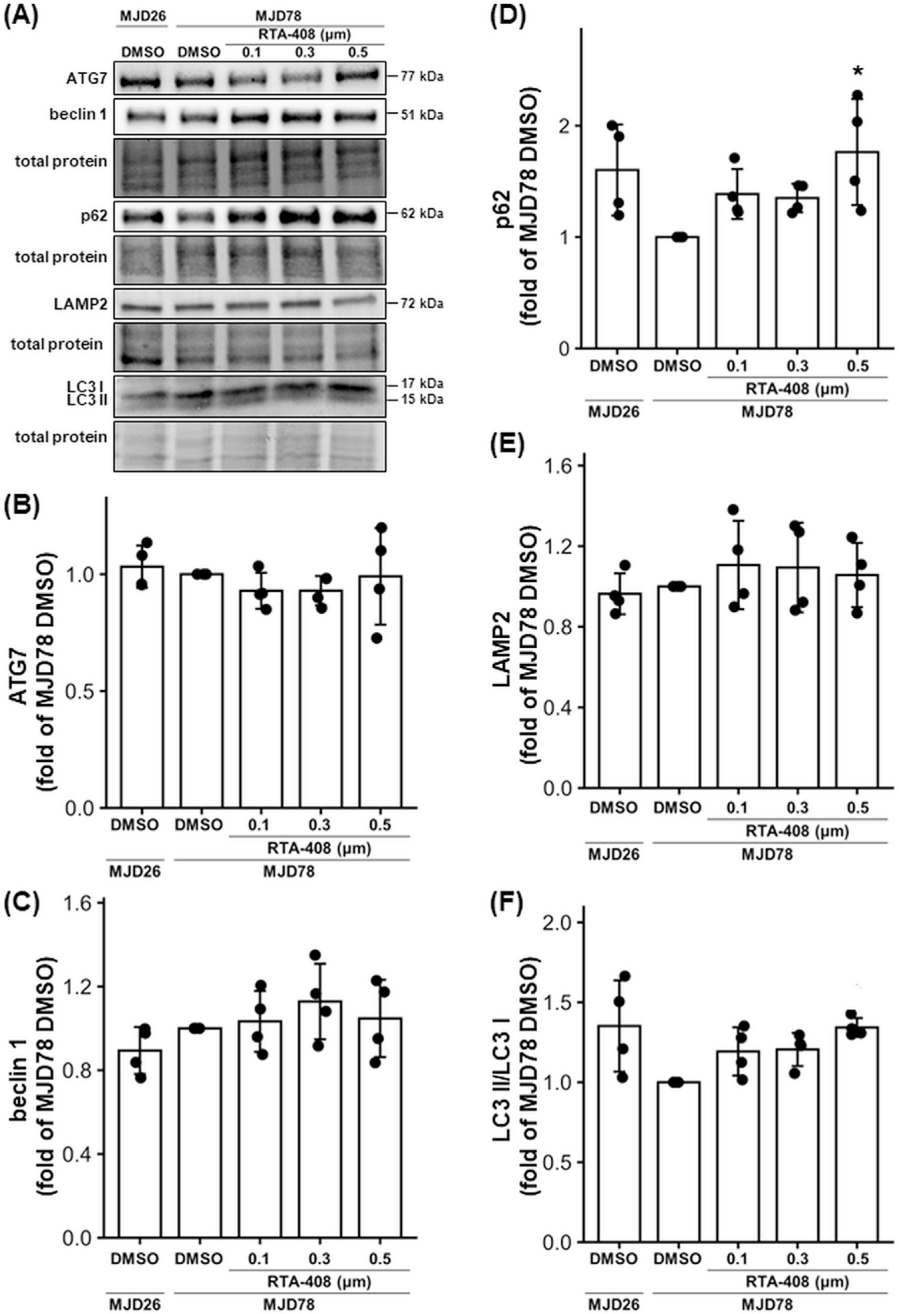
RTA-408 upregulates p62 but shows only limited changes in other autophagy-related protein readouts in MJD78 cells. **(A)** Representative western blots of ATG7, Beclin 1, p62, LAMP2 and LC3 in MJD26 and MJD78 cells treated with DMSO or RTA-408 for 24 h. Total protein was used as the loading reference. The uncropped raw Western blot images are provided in Supplementary Figure S1. **(B–F)** Quantification of the ATG7, Beclin 1, p62, LAMP2, and LC3 II/LC3 I ratio, respectively, normalised to total protein. Data are mean ± SD of 4 independent experiments. One-way ANOVA with Tukey’s HSD *post hoc* test. Asterisks indicate statistical significance vs. MJD78 + DMSO: ***: P < 0.001, **: P < 0.01, *:P < 0.05. RTA-408: omaveloxolone.

To further investigate protein clearance-related pathways, autophagy- and lysosome-associated markers were examined (Figure 2A) and quantified (Figures 2B–F). RTA-408 did not significantly alter ATG7 (Figures 2A,B) or Beclin1 expression (Figures 2A,C). In contrast, p62 expression was significantly increased at 0.5 μM (Figure 2D). LAMP2 expression (Figure 2E) and the LC3-II/LC3-I ratio (Figure 2F) were not significantly changed, although a mild restoration of LC3-II/LC3-I ratio was observed (Figure 2F). These findings indicate that RTA-408 upregulates p62 without providing definitive evidence of canonical autophagic flux activation.

### 3.3 RTA-408 increases nuclear Nrf2 and upregulates selected antioxidant proteins in MJD78 cells

**FIGURE 3:**
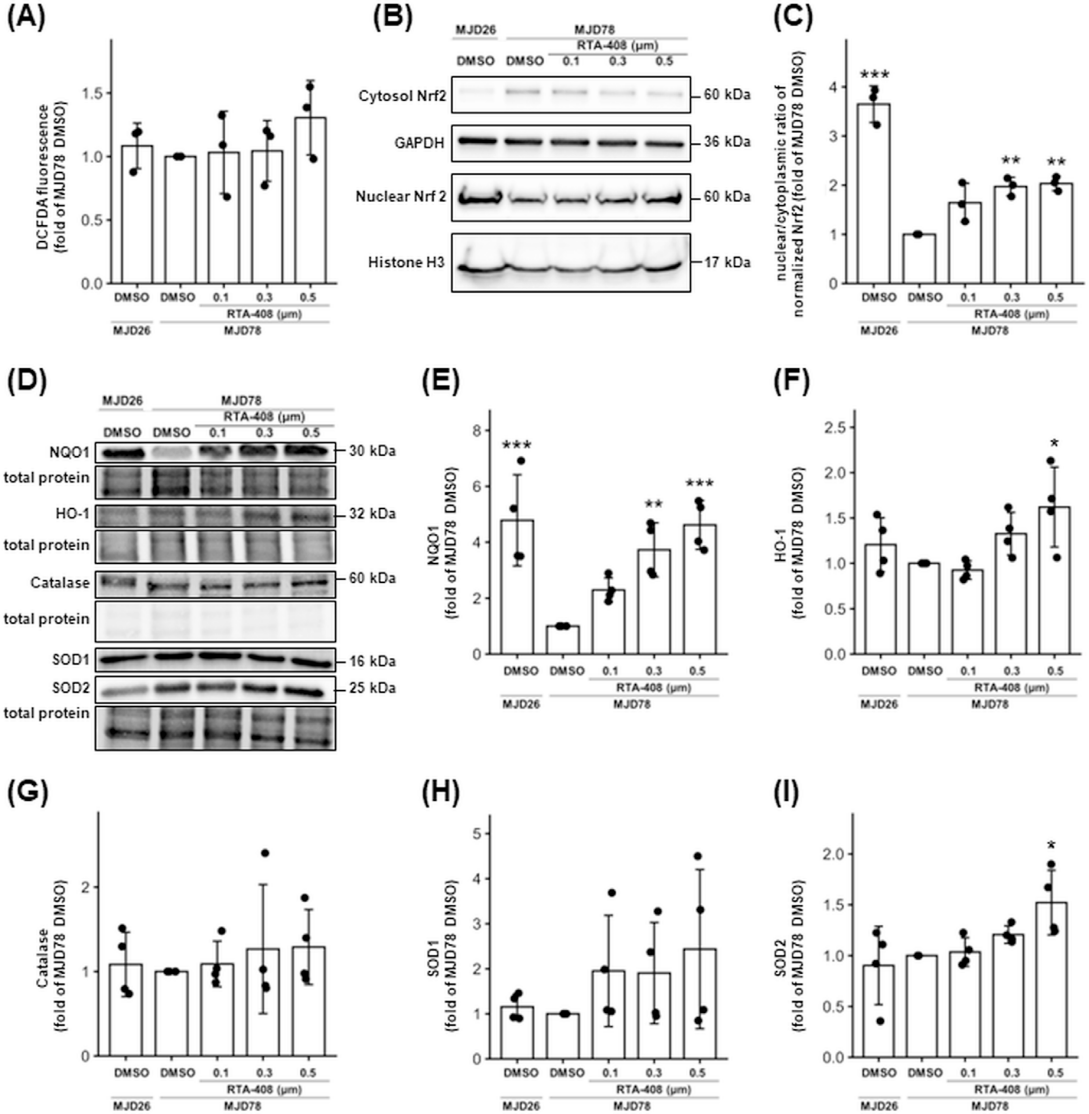
RTA-408 increases nuclear Nrf2 and selected antioxidant protein readouts in MJD78 cells. **(A)** Intracellular ROS measured by CM-H2DCFDA after 24 h of treatment with DMSO or RTA-408. **(B)** Representative western blots of cytosolic and nuclear NRF2. GAPDH and Histone H3 were used as cytosolic and nuclear loading controls, respectively. **(C)** Quantification of the nuclear/cytoplasmic Nrf2 ratio. **(D)** Representative western blots of NQO1, HO-1, Catalase, SOD1, and SOD2. Whole-cell signals were normalized to total protein. **(E–I)** Quantification of NQO1, HO-1, Catalase, SOD1, and SOD2, respectively. All uncropped raw Western blot images are provided in Supplementary Figure S1. Data are mean ± SD; intracellular ROS, n = 4; nuclear/cytoplasmic Nrf2 Western blot, n = 3; other western blots, n = 4 independent experiments. One-way ANOVA with Tukey’s HSD *post hoc* test. Asterisks indicate statistical significance vs. MJD78 + DMSO: ***: P < 0.001, **: P < 0.01, *:P < 0.05. RTA-408: omaveloxolone.

DCFDA fluorescence showed no significant difference between MJD26 and MJD78 cells under DMSO treatment, or among RTA-408–treated MJD78 groups, indicating no detectable change in intracellular ROS levels (Figure 3A). In contrast, MJD78 cells under DMSO treatment exhibited a lower nuclear-to-cytoplasmic Nrf2 ratio than MJD26 cells (Figures 3B,C). Treatment with RTA-408 increased the nuclear-to-cytoplasmic Nrf2 ratio in MJD78 cells, with more pronounced increases observed at 0.3 and 0.5 μM (Figure 3C). Western blot analysis of antioxidant proteins showed that RTA-408 increased NQO1 expression in a dose-dependent manner (Figures 3D,E). HO-1 and SOD2 levels were significantly increased at 0.5 μM RTA-408 (Figures 3F,I), whereas catalase and SOD1 levels were not significantly altered (Figures 3G,H). These results indicate that RTA-408 enhances selected Nrf2-associated antioxidant protein readouts in MJD78 cells, although this was not accompanied by a detectable reduction in intracellular ROS.

### 3.4 RTA-408 alters selected mitochondrial phenotypes without significantly reducing mitochondrial ROS

**FIGURE 4:**
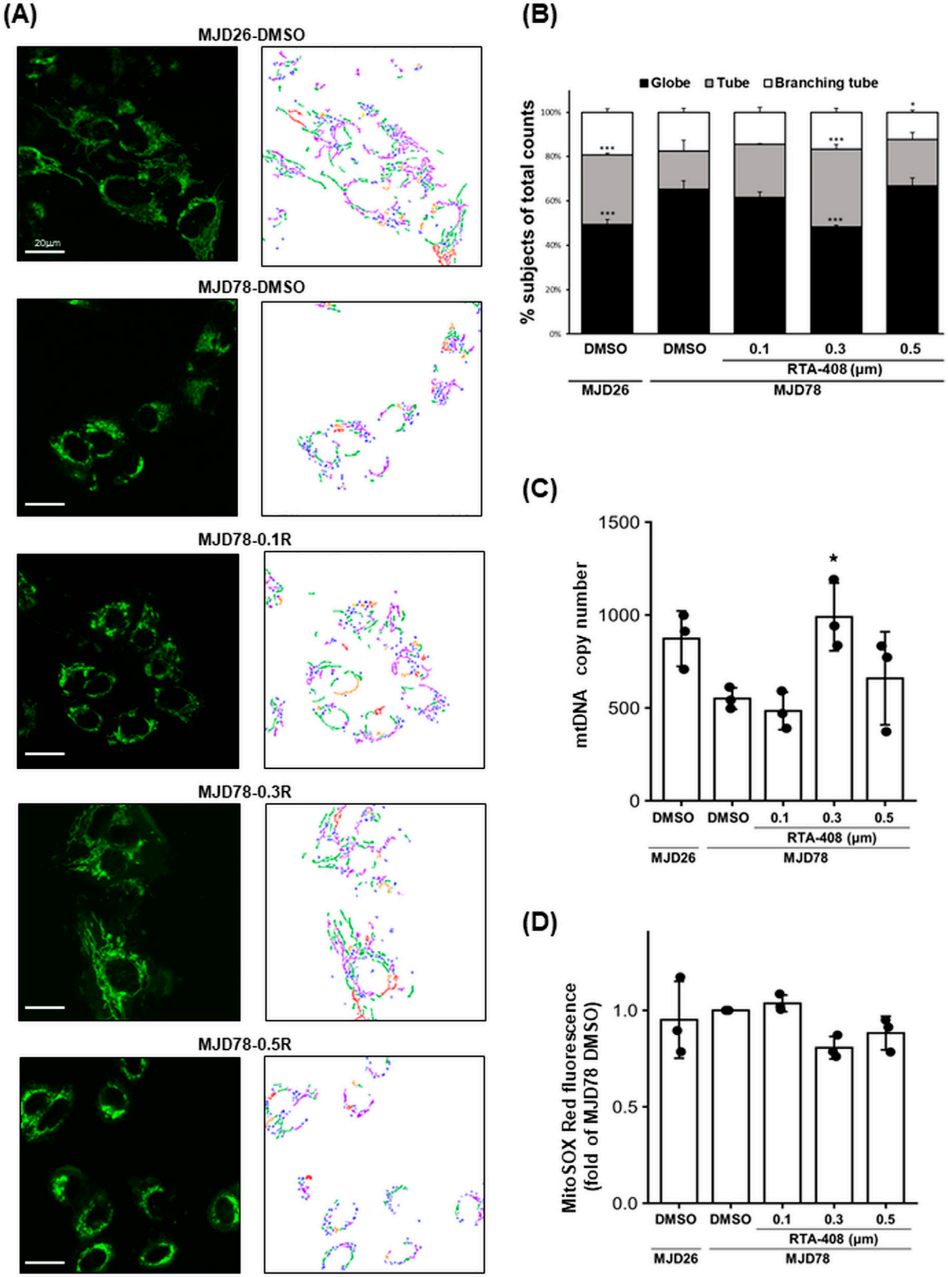
RTA-408 modulates mitochondrial morphology and increases mitochondrial DNA copy number in MJD78 cells without significantly altering mitochondrial ROS. **(A)** Representative MitoTracker Green images and corresponding color-coded mitochondrial morphology maps generated by MicroP in MJD26 and MJD78 cells treated with DMSO or RTA-408 for 24 h. In the morphology maps, globe-shaped mitochondria include swollen globes (yellow) and small globes (blue); elongated tubules include straight tubules (green); and branching tubules include branched tubules (purple), twisting tubules (orange), and loops (red). Scale bars, 20 μm. **(B)** Quantification of mitochondrial morphology distribution classified as globe, tube and branching tube forms. **(C)** Quantification of mitochondrial DNA copy number and **(D)** Quantification of mitochondrial ROS measured by MitoSOX Red fluorescence. Data are mean ± SD; morphology, n = 3 independent experiments with 3 fields per group and ∼250–350 mitochondrial objects from ∼4–6 cells per image; mtDNA copy number, n = 3; mitochondrial ROS, n = 4. One-way ANOVA with Tukey’s HSD *post hoc* test. Asterisks indicate statistical significance vs. MJD78 + DMSO: ***: P < 0.001, **: P < 0.01, *:P < 0.05. RTA-408: omaveloxolone.

Mitochondrial morphology was examined by MitoTracker Green staining and analyzed using MicroP software. In the color-coded MicroP images, globe-shaped mitochondria included swollen globes in yellow and small globes in blue; elongated tubules included straight tubules in green; and branching tubules included branched tubules in purple, twisting tubules in orange, and loops in red (Figure 4A). Representative images showed differences in mitochondrial morphology between MJD26 and MJD78 cells. Compared with MJD26 cells, MJD78 cells showed a higher proportion of globe-shaped mitochondria and a lower proportion of elongated tubule-shaped mitochondria (Figure 4B). RTA-408 treatment partially altered the mitochondrial morphology distribution in MJD78 cells. At 0.3 μM, RTA-408 significantly reduced globe-shaped mitochondria and increased elongated tubule-shaped mitochondria compared with MJD78 DMSO cells. At 0.5 μM, RTA-408 significantly reduced branching tubule-shaped mitochondria (Figure 4B).

Compared with MJD26 cells, MJD78 cells showed a lower mtDNA copy number, although the difference did not reach statistical significance. Treatment with 0.3 μM RTA-408 significantly increased mtDNA copy number in MJD78 cells compared with MJD78 DMSO cells (Figure 4C). MitoSOX Red fluorescence did not differ significantly between MJD26 and MJD78 cells or among RTA-408–treated MJD78 groups (Figure 4D). These results suggest that RTA-408 affects selected mitochondrial phenotypes, particularly mitochondrial morphology distribution and mtDNA copy number, but do not establish improved mitochondrial function.

### 3.5 RTA-408 improves survival and locomotor performance in the SCA3 fly model

In the initial stage of the study, male and female flies were examined separately. Male flies showed a shorter lifespan than female flies in both Q27 and Q78 backgrounds, which limited the detection of stable survival differences. Therefore, female flies were used in subsequent fly experiments.

**FIGURE 5:**
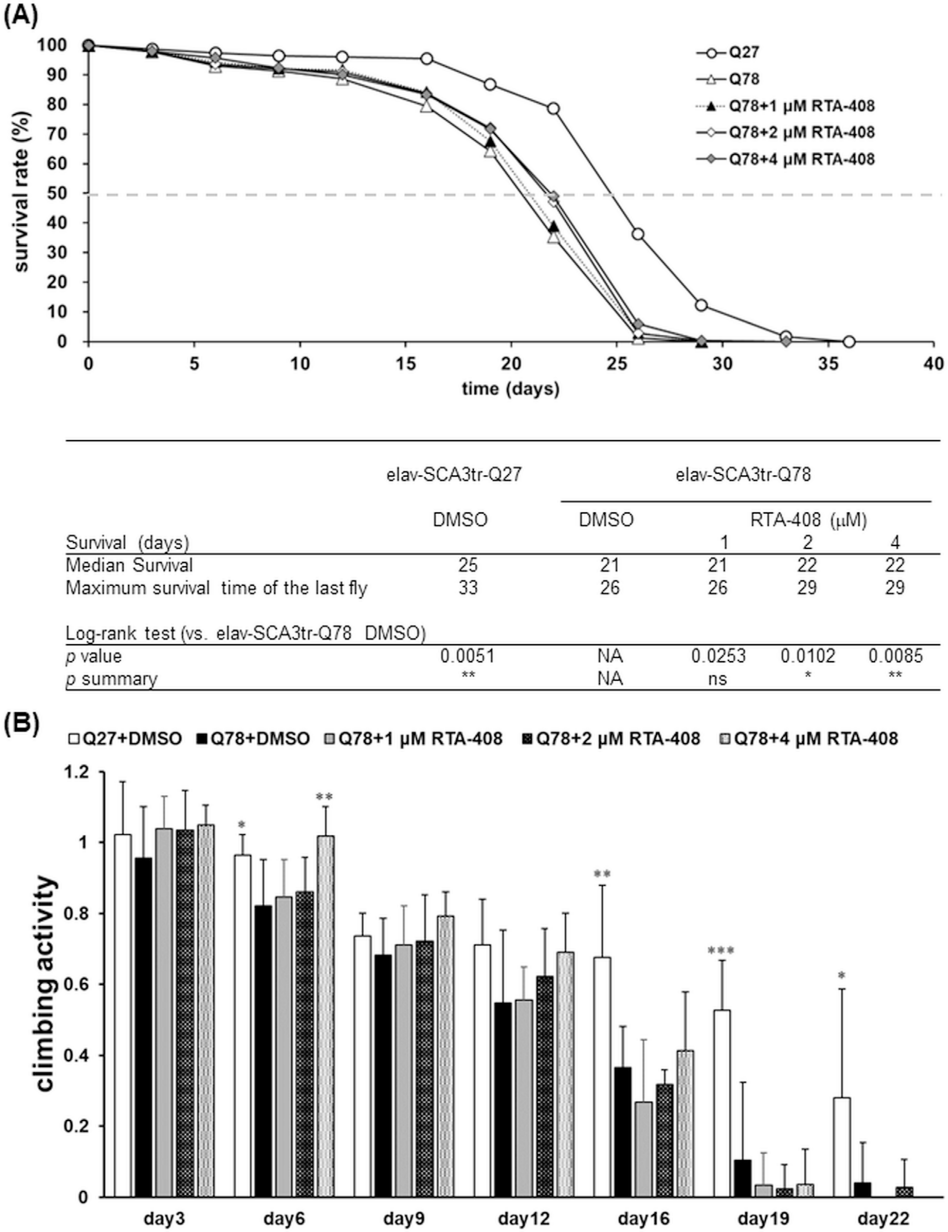
RTA-408 partially improves survival and locomotor decline in female elav-SCA3tr-Q78 flies. **(A)** Kaplan–Meier survival curves and summary table for female elav-SCA3tr-Q27 and elav-SCA3tr-Q78 flies treated with DMSO or RTA-408 (1, 2 or 4 μM). Each group initially contained 350 flies distributed into 10 vials (35 flies/vial). Survival was analyzed by log-rank test. **(B)** Longitudinal climbing activity measured every 3 days by negative geotaxis assay. In each batch, 35 flies per group were tested; each session was performed in triplicate, and the highest value was used for analysis. A total of 8 independent batches were analyzed. Climbing activity was defined as the proportion of flies crossing the 5 cm mark within 18 s; proportional data were arcsine square-root transformed before analysis. Data are mean ± SD. Asterisks indicate statistical significance vs. Q78 + DMSO: ***: P < 0.001, **: P < 0.01, *:P < 0.05. RTA-408: omaveloxolone.

In female elav-SCA3tr-Q78 flies, survival was reduced compared with elav-SCA3tr-Q27 control flies. The median survival was 25 days in Q27 flies and 21 days in Q78 flies. RTA-408 treatment partially improved survival in Q78 flies, with median survival extended to 22 days at 2 and 4 μM. The maximum survival was also increased from 26 days in Q78 DMSO flies to 29 days in the 2 and 4 μM RTA-408 groups. Log-rank analysis showed significant differences in the 2 μM and 4 μM RTA-408 groups compared with Q78 DMSO flies, whereas the 1 μM group did not reach statistical significance (Figure 5A).

In the climbing assay, Q78 flies showed progressive locomotor decline over time compared with Q27 controls. RTA-408 treatment partially improved climbing performance, with the most evident effect observed in the 4 μM group, particularly at day 6 and day 12. At later time points, climbing activity declined markedly in Q78 flies, and the treatment effect became less evident (Figure 5B). The reduced treatment effect at later time points suggests that RTA-408 produced a partial and stage-dependent benefit rather than sustained rescue throughout disease progression.

### 3.6 RTA-408 ameliorates external eye degeneration in the SCA3 fly model

**FIGURE 6:**
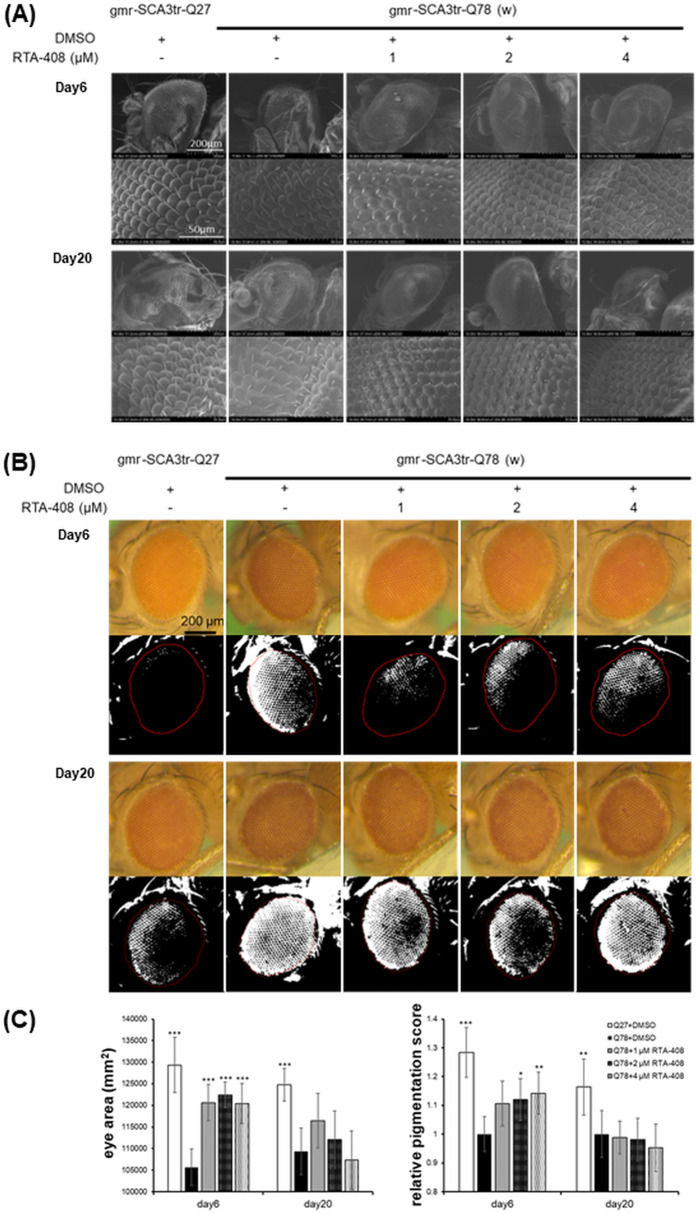
RTA-408 partially ameliorates external eye degeneration in female gmr-SCA3tr-Q78 flies. **(A)** Representative scanning electron microscopy (SEM) images of adult eyes from female gmr-SCA3tr-Q27 and gmr-SCA3tr-Q78 flies treated with DMSO or RTA-408 (1, 2, or 4 μM) on day 6 and day 20 after eclosion. Scale bars: 200 μm for low-magnification images and 50 μm for high-magnification images. **(B)** Representative bright-field images and corresponding processed images used for pigmentation analysis on day 6 and day 20. Scale bar: 200 μm. **(C)** Quantification of eye area and relative pigmentation score on day 6 and day 20 after eclosion. Data are mean ± SD; eye area and pigmentation score, 8 eyes/group; SEM, 3 eyes/group. One-way ANOVA with Tukey’s HSD *post hoc* test. Asterisks indicate statistical significance vs. Q78 + DMSO: ***: P < 0.001, **: P < 0.01, *:P < 0.05. RTA-408: omaveloxolone. SEM images were used for qualitative morphological observation rather than quantitative analysis.

SEM provided qualitative support for external eye degeneration in Q78 flies, which tended to show a more irregular ommatidial arrangement and less preserved ommatidial morphology than Q27 controls. RTA-408–treated flies appeared to show partial preservation of ommatidial organization compared with Q78 + DMSO flies, but because SEM analysis was limited to three eyes per group, these observations were interpreted descriptively rather than quantitatively (Figure 6A). Bright-field images and corresponding binary/outlined images were used to assess external eye size and pigmentation (Figure 6B). Quantitative analysis showed that, on day 6, Q78+DMSO flies had significantly smaller eye areas than Q27+DMSO controls. RTA-408 treatment at 1, 2, and 4 μM significantly increased eye area compared with Q78+DMSO flies, indicating partial rescue of the early eye size defect (Figure 6C). Q78+DMSO flies also showed reduced pigmentation compared with Q27+DMSO controls on day 6. RTA-408 treatment at 2 and 4 μM significantly increased the pigmentation score, whereas 1 μM did not show a significant effect (Figure 6C). On day 20, Q78+DMSO flies continued to show reduced eye area and pigmentation compared with Q27+DMSO controls. However, RTA-408 treatment at 1, 2, or 4 μM did not significantly improve eye area or pigmentation at this later time point (Figure 6C). These results show that RTA-408 partially ameliorates early external eye degeneration in SCA3tr-Q78 flies, with clearer effects on day 6 than on day 20.

## 4 Discussion

This study demonstrated that RTA-408 exerts protective effects in SCA3-related cellular and *Drosophila* models. In MJD78 cells, RTA-408 improved cell viability, reduced apoptosis, and decreased detectable mutant ataxin-3 accumulation. These cellular effects were accompanied by an increased nuclear-to-cytoplasmic Nrf2 ratio, upregulation of selected Nrf2-associated antioxidant proteins, including NQO1, HO-1, and SOD2, and increased p62 expression. However, intracellular ROS, mitochondrial ROS, and most canonical autophagy-related readouts were not significantly altered. Therefore, the present findings should be interpreted as protective effects associated with Nrf2-related antioxidant protein responses and p62 upregulation, rather than as definitive evidence of Nrf2-dependent protection or canonical autophagic flux activation (Ulasov et al., 2022).

At the proteostasis level, polyQ-expanded ataxin-3 readily forms aggregation-prone species and disrupts cellular protein quality-control pathways. Fragments derived from expanded ataxin-3 can escape cytoplasmic protein quality-control mechanisms, and aggregate formation may further impair efficient proteasomal degradation of polyglutamine proteins and related substrates (Haacke et al., 2006; Ulasov et al., 2022; Verhoef et al., 2002; Menzies et al., 2010). In this context, the combination of increased p62 expression, reduced detectable mutant ataxin-3 accumulation, and unchanged normal ataxin-3 levels is noteworthy. p62 is an Nrf2-responsive protein and a selective autophagy adaptor implicated in the handling of aggregation-prone proteins (Saitoh et al., 2015; Breuer et al., 2010; Zhou et al., 2014). However, because ATG7, Beclin 1, LAMP2, and LC3-II/LC3-I were not significantly increased, the present data do not establish enhanced autophagic degradation. The difference in LC3-II/LC3-I between the 0.3 μM and 0.5 μM groups further suggests that autophagy-related readouts may respond nonlinearly to RTA-408 concentration; therefore, the effect observed at the higher concentration may reflect additional stress-related or compensatory responses rather than straightforward activation of autophagic flux. Alternative explanations should be considered, including Nrf2-driven p62 transcription, impaired p62 turnover, altered mutant ataxin-3 solubility, sequestration, or altered protein synthesis. Future studies using autophagic flux assays, soluble/insoluble ataxin-3 fractionation, p62 gain- and loss-of-function experiments, and p62–ataxin-3 colocalization will be required to determine whether p62 directly contributes to mutant ataxin-3 handling (Saitoh et al., 2015; Zhou et al., 2014).

RTA-408 also increased Nrf2 nuclear accumulation and upregulated selected antioxidant proteins in MJD78 cells. The increased cytosolic Nrf2 level together with a reduced nuclear/cytoplasmic Nrf2 ratio in MJD78 cells may suggest impaired nuclear translocation or cytoplasmic retention of Nrf2 under mutant ataxin-3–associated stress. Thus, RTA-408 may partially restore Nrf2 nuclear accumulation rather than simply increasing total Nrf2 abundance. NQO1 increased in a dose-dependent manner, whereas HO-1 and SOD2 were significantly increased at 0.5 μM. In contrast, catalase and SOD1 were not significantly altered. These findings show that RTA-408 enhances selected Nrf2-associated antioxidant protein responses in MJD78 cells. Importantly, previous work using the same SK-N-SH-MJD78 cellular model showed that RTA-408 activates ARE/Nrf2 transcriptional activity and that Nrf2 siRNA attenuates RTA-408/JM17-associated antioxidant and mutant ataxin-3-related readouts (Wu et al., 2022). Other SCA3 cell and *Drosophila* studies using Nrf2-related interventions, including caffeic acid/resveratrol and erinacine A-enriched Hericium erinaceus extract, further support the biological relevance of Nrf2-related responses in SCA3 models (Wu et al., 2018; Wu et al., 2024). However, the present study did not repeat Nrf2 knockdown experiments; therefore, the Nrf2-related changes observed here should be interpreted as pharmacodynamic and literature-supported readouts accompanying RTA-408 treatment rather than as *de novo* proof of Nrf2 dependency. Moreover, these molecular changes were not accompanied by a significant reduction in DCFDA fluorescence or MitoSOX Red fluorescence. This discrepancy suggests that RTA-408 may increase antioxidant response capacity without necessarily reducing steady-state measurable intracellular or mitochondrial ROS under the present experimental conditions. One possible explanation is that Nrf2 activation may increase the buffering capacity and inducible antioxidant reserve of MJD78 cells rather than lowering basal ROS levels under unstimulated culture conditions. In addition, if ROS production and antioxidant clearance are simultaneously altered, steady-state ROS fluorescence may remain unchanged despite increased expression of antioxidant defense proteins. DCFDA and MitoSOX Red may also fail to capture compartment-specific, transient, or low-amplitude oxidative changes. Thus, the present data support Nrf2-associated antioxidant responses but do not establish ROS suppression as the direct mechanism of protection.

In addition to antioxidant- and proteostasis-related changes, RTA-408 altered mitochondrial morphology distribution and increased mtDNA copy number in MJD78 cells. Notably, the mitochondrial readouts did not follow the same dose-dependent pattern as the Western blot results. The most evident mitochondrial changes were observed at 0.3 μM, particularly in mitochondrial morphology distribution and mtDNA copy number, whereas 0.5 μM showed stronger effects on p62 and selected antioxidant proteins. These findings indicate that mitochondrial morphology and mtDNA copy number may have distinct dose sensitivity to RTA-408. Because mitochondrial ROS was not significantly altered and mitochondrial functional assays such as ATP production, oxygen consumption rate, membrane potential, and fusion/fission markers were not included in the final analysis, these results should be interpreted as selected mitochondrial phenotypic changes rather than definitive restoration of mitochondrial function.

The fly experiments were designed to evaluate whether RTA-408 could slow post-eclosion disease progression rather than prevent disease onset. Because disease-related pathology is already detectable during larval development in gmr- or elav-driven *Drosophila* MJD models, including abnormalities in developing eye tissues and the nervous system (Robinow and White, 1988; Warrick et al., 1998), the post-eclosion intervention used here is particularly relevant for assessing therapeutic rather than preventive efficacy. Consistent with this design, the *Drosophila* experiments further supported the *in vivo* protective potential of RTA- 408. In female elav-SCA3tr-Q78 flies, RTA-408 partially improved survival and locomotor performance. Median survival was modestly extended at selected doses, and climbing activity showed partial improvement, particularly during earlier time points. Previous studies showed that elav-SCA3tr-Q78 flies expressing polyQ-expanded mutant ataxin-3 exhibit a markedly shorter post-eclosion lifespan than elav-SCA3tr-Q27 flies expressing normal ataxin-3, supporting this model as a progressive adult neurodegenerative phenotype (Wu et al., 2018). In the gmr-SCA3tr-Q78 eye model, RTA-408 partially ameliorated external eye degeneration. On day 6, RTA-408 increased eye area at 1, 2, and 4 μM and improved pigmentation at 2 and 4 μM. However, these effects were not maintained on day 20. Previous studies have shown that gmr-MJDtr-Q78 flies exhibit early developmental eye defects, including rough surface, irregular ommatidial arrangement, and pigment degeneration (Saitoh et al., 2015). These findings suggest that RTA-408 may exert greater benefit during early-to-mid disease stages, whereas its effect becomes limited when disease burden and ageing-related stress accumulate. Furthermore, SEM further showed partial preservation of ommatidial organization in RTA-408–treated flies compared with Q78 DMSO flies. However, because SEM analysis was limited by sample number and image quality, these observations should be interpreted descriptively rather than as definitive quantitative evidence. Therefore, the fly data more strongly support a partial disease-modifying effect of RTA-408 based on survival, locomotor activity, eye area, and pigmentation analyses, especially during earlier stages of phenotypic progression.

The *Drosophila* models used in this study provide a genetically tractable *in vivo* system for evaluating polyQ-expanded ataxin-3 toxicity, survival, locomotor decline, and eye degeneration. Although these models do not reproduce human cerebellar anatomy or mammalian pharmacokinetics, they are useful for testing whether candidate compounds can ameliorate conserved proteotoxicity-related and neurodegeneration-related phenotypes *in vivo*. Therefore, the fly data in the present study should be interpreted as exploratory *in vivo* phenotypic support for the protective potential of RTA-408 rather than pathway-level validation in the *Drosophila* model. Although previous studies support the relevance of Nrf2 activation and Nrf2 knockdown in SCA3 cellular models and RTA-408-related responses, CncC/Nrf2 target gene expression was not examined in the present fly cohort. Accordingly, the mechanistic interpretation of the present study is based primarily on cellular Nrf2-related readouts and prior evidence from the same SCA3 cellular model, whereas the *Drosophila* experiments provide organism-level phenotypic support. Further mammalian validation will be important for clarifying the translational relevance of RTA-408 in SCA3.

In summary, RTA-408 reduced detectable mutant ataxin-3 accumulation, improved cell viability, and reduced apoptosis in MJD78 cells. These effects were associated with increased Nrf2 nuclear accumulation, selective upregulation of antioxidant proteins, increased p62 expression, and changes in mitochondrial morphology and mtDNA copy number. In *Drosophila* models, RTA-408 partially improved survival, locomotor performance, and early external eye degeneration. Together, these findings support the protective potential of RTA-408 in SCA3-related models. Several limitations should be noted. First, Nrf2 dependency was not directly tested using knockdown, pharmacological inhibition, ARE reporter assays, or rescue experiments. Second, autophagic flux was not directly assessed using lysosomal inhibitors, and the physical interaction or colocalization between p62 and mutant ataxin-3 was not examined. Third, pathway-level validation was not performed in the *Drosophila* cohort, and CncC/Nrf2 target gene expression after RTA-408 treatment remains to be determined. Fourth, mitochondrial functional assays, including ATP production, oxygen consumption rate, membrane potential, and fusion/fission markers, were not included in the final analysis. Finally, dedicated toxicological, pharmacokinetic, food-stability, and long-term safety evaluations of RTA-408 in SCA3 models were not performed. Further studies in mammalian models are warranted to clarify its mechanisms, therapeutic window, and translational relevance for SCA3.

## 5 Conclusion

In conclusion, RTA-408 exerted protective effects in cellular and *Drosophila* models of SCA3. In MJD78 cells, RTA-408 reduced detectable mutant ataxin-3 accumulation, improved cell viability, reduced apoptosis, increased Nrf2 nuclear accumulation, upregulated selected antioxidant proteins, increased p62 expression, and altered mitochondrial morphology and mtDNA copy number. In SCA3 *Drosophila* models, RTA-408 partially improved survival, locomotor performance, and early external eye degeneration. Together, these findings support further evaluation of RTA-408 as a candidate therapeutic strategy for SCA3, while additional mechanistic, toxicological, and mammalian validation studies are required.

## Data Availability

The original data presented in this study are included in the article/Supplementary Material. Further inquiries can be directed to the corresponding author.
