# Supplementary material for: Therapeutic potential of the Nrf2 activator omaveloxolone in spinocerebellar ataxia type 3 in cellular and Drosophila models
Source: Front Pharmacol. 2026 Aug 5;17:1872142. doi: 10.3389/fphar.2026.1872142 (PMC13487206; doi:10.3389/fphar.2026.1872142)
Supplement: Supplementary file 1 [file Supplementaryfile1.docx]

Supplementary Material

# 1 Supplementary results

List of primary antibodies used for immunoblotting and related protein analyses is provided in Table S1. Uncropped raw western blot images corresponding to the immunoblotting data presented in Figures 1–3 are provided in Figure S1. The images include raw blots for mutant and normal ataxin-3 (Figure S1A), autophagy- and lysosome-related proteins, including ATG7, Beclin 1, p62/SQSTM1, LAMP2, and LC3 (Figure S1B), and NRF2 signaling proteins and antioxidant enzymes, including NRF2, NQO1, HO-1, catalase, SOD1, and SOD2 (Figure S1C). Total protein staining, GAPDH, Histone H3, or Lamin B1 were used as loading or fractionation controls where applicable. Molecular weight markers, target protein sizes, experimental groups, and biological replicates are indicated in each panel. The first blot image in each set represents the cropped representative blot shown in the corresponding main figure. Red boxes indicate the regions selected for presentation in the main figures, and arrows indicate the positions of the target proteins in all panels.

# 2 Supplementary Table S1

# Table S1. List of primary antibodies used for immunoblotting and related protein analyses.

| **Target** | **Vendor** | **Catalog no.** | **RRID** | **Host species** |
| --- | --- | --- | --- | --- |
| Ataxin-3 | Abcam | ab175265 | AB_2940952 | Rabbit |
| Beclin 1 | Novus Biologicals | NB110-87318 | AB_1201249 | Rabbit |
| ATG7 | Abcam | ab133528 | AB_2532126 | Rabbit |
| LAMP2 | Thermo Fisher Scientific | PA1-655 | AB_2134625 | Rabbit |
| p62/SQSTM1 | Sigma-Aldrich | P0067 | AB_1841064 | Rabbit |
| LC3B | Proteintech | 18725-1-AP | AB_2137745 | Rabbit |
| HO-1 | Millipore | 374090 | AB_2248409 | Rabbit |
| SOD2 | GeneTex | GTX116093 | AB_10624558 | Rabbit |
| SOD1 | GeneTex | GTX100659 | AB_1951972 | Rabbit |
| Catalase | Abcam | ab16731 | AB_302482 | Rabbit |
| NRF2 | Santa Cruz Biotechnology | sc-365949 | AB_10917561 | Mouse |
| NQO1 | Abcam | ab28947 | AB_881738 | Mouse |
| GAPDH | Millipore | ABS16 | AB_10806772 | Rabbit |
| Lamin B1 | Abcam | ab16048 | AB_443298 | Rabbit |
| Histone H3 | Abcam | ab1791 | AB_302613 | Rabbit |

# 3  Supplementary Figure S1

# Figure S1. Uncropped raw western blot images corresponding to Figures 1–3. The images include raw blots for mutant and normal ataxin-3 (A), autophagy- and lysosome-related proteins including ATG7, Beclin 1, p62/SQSTM1, LAMP2, and LC3 (B), and NRF2 signaling proteins and antioxidant enzymes including NRF2, NQO1, HO-1, catalase, SOD1, and SOD2 (C). Total protein staining, GAPDH, Histone H3, or Lamin B1 were used as loading or fractionation controls where applicable. Molecular weight markers, target protein sizes, experimental groups, and biological replicates are indicated in each panel. The first blot image in each set represents the cropped representative blot shown in the corresponding main figure. Red boxes indicate the regions selected for presentation in the main figures, and arrows indicate the positions of the target proteins in all panels.
